# Influence of Supplementing *Sesbania grandiflora* Pod Meal at Two Dietary Crude Protein Levels on Feed Intake, Fermentation Characteristics, and Methane Mitigation in Thai Purebred Beef Cattle

**DOI:** 10.3390/vetsci8020035

**Published:** 2021-02-23

**Authors:** Narirat Unnawong, Anusorn Cherdthong, Sarong So

**Affiliations:** Tropical Feed Resources Research and Development Center (TROFREC), Department of Animal Science, Faculty of Agriculture, Khon Kaen University, Khon Kaen 40002, Thailand; nariratunnawong@kkumail.com (N.U.); sarong07so@gmail.com (S.S.)

**Keywords:** *Sesbania grandiflora*, tannins, saponin, methane, fecal nitrogen, ammonia, propionate

## Abstract

The aim of the study was to evaluate the effect of crude protein (CP) levels in concentrate and *Sesbania grandiflora* pod meal (SG) supplementation on feed intake, rumen fermentation, and methane (CH_4_) mitigation in Thai purebred beef cattle. Four cattle with 100 ± 5.0 kg body weight were used in this study. A 2 × 2 factorial experiment in a 4 × 4 Latin square design was conducted, in which factor A was the CP levels in concentrate of 14% and 16% of dry matter (DM) and factor B was the supplement levels of SG at 0.4% and 0.6% DM intake, respectively. The results showed that the CP content in concentrate and SG supplementation had no interaction effect on intake, digestibility, ruminal ecologies, ruminal fermentation products, and nitrogen utilization. Increasing CP content to 16% significantly (*p* < 0.05) increased the ruminal ammonia nitrogen (NH_3_-N), nitrogen (N) intake, N absorption, and N retention. SG supplementation significantly (*p* < 0.05) decreased CP digestibility, NH_3_-N, blood urea nitrogen, and protozoa. In addition, SG significantly decreased acetate (C2), acetate to propionate ratio, methane, and fecal N excretion, while it significantly increased total volatile fatty acids (VFAs) and propionate (C3) concentration. In conclusion, SG could mitigate methane emission and enhance nitrogen utilization.

## 1. Introduction

The emission of methane (CH_4_) is labeled as a greenhouse gas, which is one of the hot environmental problems [1]. Besides its influence on the environment, CH_4_ is also responsible for the energy loss in ruminants from ingested feeds, up to 12% of gross energy intake [2]. Thus, mitigating CH_4_ emission not only benefits the environment, but also enhances the energy utilization in ruminants. Various approaches including dietary manipulation, antibiotics, and plant secondary compounds (PSCs) for CH_4_ mitigation have been tested, and using PSCs such as saponins and tannins has been shown to be the most effective approach recently [3,4,5] for CH_4_ mitigation. Saponins affect CH_4_ mitigation by lowering the protozoa population and changing volatile fatty acid (VFA) production patterns, as well as enhancing fiber degradation [6]. Tannins indirectly and directly affect methanogenesis, resulting in lower CH_4_ production. In addition, tannins can protect protein metabolism in the rumen by tannin-protein binding [7], which may enhance protein metabolism in the small intestine. However, the effect of saponins and tannins varies depending on source, molecular weight, temperature, soil quality, nutrient stress, and topography [8,9]. *Sesbania grandiflora* (*S. grandiflora*) is a tree classed in the *Fabaceae* family and *Sesbania* genus. The *S. grandiflora* pods contain 35% of crude protein (CP) and PSCs including tannins, flavonoids, steroids, and triterpenes [10,11]. Jayanegara et al. [9] reported that *Sesbania sesban* (*S. sesban*) leaves significantly mitigated in vitro CH_4_ production when used as a sole substrate and added to concentrate-based diets. However, the effect of *S. grandiflora* pod meal (SG) containing saponins and tannins with different dietary CP levels has not yet been evaluated. Increasing protein content in concentrate might be prevented from ruminal microbial degradation when SG containing tannins increase.

Two hypotheses were made: (1) CP and SG have no interaction effect on intake, digestibility, rumen fermentation, CH_4_ production, and nitrogen utilization and (2) the effect of SG supplementation is affected by CP level in concentrate. Therefore, the aim of the study was to evaluate the effect of CP content in concentrate and SG supplementation containing saponins and tannins on feed intake, rumen fermentation, and CH_4_ mitigation in Thai purebred beef cattle.

## 2. Materials and Methods

Approval no. IACUC-KKU-114/62 was issued by the Animal Ethics Committees of Khon Kaen University.

### 2.1. S. grandiflora Pods Meal

Fresh *S. grandiflora* pods were collected from Udon-Thani and Khon Kaen provinces (Thailand) from September to November 2020. The pods were sun-dried for 2 to 3 weeks and ground through a 1 mm sieve (Cyclotech Mill, Tecator, Höganäs, Sweden). The SG sample was analyzed for saponin and tannin content. The analysis procedure was modified from Kwon et al. [12] and Edeoga et al. [13]. In brief, 5 g of SG was put into an Erlenmeyer flask with 80% methanol added. Then, the flask contents were evaporated using a microwave for 30 min and transferred through Whatman No. 41 into a new flask. The same process was repeated four times. After filtering, the sample was evaporated using a rotary vacuum evaporator to obtain the final volume of approximately 25 mL. The obtained solution was separated with 99.9% ether using a separatory funnel. Then, the residue at the bottom-funnel was re-separated with Butanol-N and washed two times using 5% NaCl. After washing, the residue was heated at 80 °C for 30 min in a water bath. The crude saponin content of SG was obtained after the residue was oven-dried overnight at 60 °C. The tannin content was analyzed by modifying the method of Burns [14]. The chemical composition of SG was analyzed according to Association of Official Agricultural Chemists (AOAC) [15] including dry matter (DM, ID 967.03), organic matter (OM, ID 942.05), CP (ID 984.13), ether extract (EE, ID 920.39), and acid detergent fiber (ADF), and Van Soest et al.’s [16] method for neutral detergent fiber (NDF). The crude saponin and tannin content and chemical composition of the SG are shown in Table 1.

### 2.2. Cattle, Design, and Feeding

Four Thai purebred beef cattle of 100 ± 5.0 kg initial body weight (BW) were used in this study. A 2 × 2 factorial experiment in a 4 × 4 Latin square design was conducted, in which factor A was the CP content in concentrate of 14% and 16% of DM and factor B was the supplement content of SG at 0.4% and 0.6% DM intake. Cattle were placed in an individual pen with free access to a mineral block and clean water and fed dietary treatments at 07:00 and 16:00. Four periods of 14 days were used for treatment adaptation, and 7 days were used for sample collection and digestibility study. The concentrate was fed at 1.0% BW, and rice straw (RS) was used as an exclusive roughage source and provided ad libitum. The chemical composition of concentrate and RS and rations is provided in Table 1 and Table 2.

### 2.3. Sample Collection and Sampling Procedures

The offered feeds including concentrate and RS and the remaining portions were recorded daily during the experiment. The cattle were transferred to metabolism crates and stayed there for 7 days. The samples of concentrate and RS and their remaining portions were collected and separated (two parts); the first part of the samples was analyzed for DM content and the remaining part of the samples was pooled and frozen by cattle and period for chemical composition analysis. Fecal and urine samples were collected for 7 days using the total collection method to study the digestibility and nitrogen balance. Five percent fecal samples of total fresh weight were withdrawn and separated (two parts); the first part of fecal samples was analyzed for DM content, and the remaining part of fecal samples was pooled and frozen by cattle and period for chemical composition analysis. The frozen samples including feeds (concentrate and RS), refusals (concentrate and RS), and feces were thawed, oven-dried at 60 °C, and ground through a 1 mm sieve for chemical composition analysis including DM, ash, CP, and ADF, following the AOAC [15] method, and NDF according to Van Soest et al.’s [16] method. The frozen urine samples were thawed and analyzed for urinary nitrogen using the Kjeldahl method according to AOAC [15].

On day 21 of each period, 10 mL of blood samples was collected from the jugular vein of each animal at 0 and 4 h after feeds were offered. The samples of blood were stored in ethylenediaminetetraacetic acid (EDTA) as an anticoagulant, and plasma was obtained using a centrifuge (500× *g* rpm, 10 min, 4 °C) and then analyzed for blood urea nitrogen (BUN) [17]. The fluid (100 mL) from the rumen was withdrawn from each animal via a stomach tube attached to a vacuum pump. A portable pH meter (HANNA, HI 8424, Hanna Instruments, Kallang Way, Singapore) was used for the fluid pH measurement. The fluid samples were passed through cheesecloth (four layers) and separated (two parts); the first part (45 mL fluid) was mixed with H_2_SO_4_ (5 mL) at a ratio of 1:9. The clear sample solutions were obtained via centrifuge (16,000× *g*, 15-min), and then analyzed for NH_3_-N (Kjeltech Auto 1030 analyzer, EquipNet Thailand, Bangkok, Thailand) and VFA proportions (acetate—C2, propionate—C3, butyrate—C4) using high-performance liquid chromatography [18]. The VFA proportion concentrations were calculated for the CH_4_ concentration. The stoichiometrical model used for estimating CH_4_ from VFA composition followed Moss et al.’s [19] equation: CH_4_ = 0.45 (C2) − 0.275 (C3) + 0.4 (C4). Even though the determination of CH_4_ production is usually achieved using a respiratory chamber or the gas chromatography technique, this is unfortunately very costly, and such facilities may not be available, especially in developing countries. Thus, the calculation of CH_4_ production from VFA profiles is expected to be a solution to the problem. The remaining part was mixed in 10% formalin at a ratio of 1:9 (1 mL ruminal fluid and 9 mL formalin) for protozoal population study using a direct count technique under a microscopic (Boeco, Hamburg, Germany).

### 2.4. Calculations and Statistical Analysis

All data were analyzed according to a 2 × 2 factorial in a 4 × 4 Latin square design using a generalized linear model (GLM) procedure. The model is as follows:

Y_ijkl_ = µ + α_i_ + β_j_ + αβ_ij_ + A_k_ + P_l_ + ε_ijkl_ (1)

where Y_ijk_ is the observation, µ is the overall mean, α_i_ is the effect of CP content at 14% and 16%, β_j_ is the effect of SG supplementation at 0.4% and 0.6%, αβ_ij_ is the interaction effect between CP content and SG supplementation, A_j_ is the effect of the animal, P_k_ is the effect of the period, and ε_ijkl_ is the residual effect. Differences between treatment combination means were tested using Tukey test [20] and *p* < 0.05 was used to declare the level of statistical difference.

## 3. Results

### 3.1. Chemical Composition of Diets

The concentrate was formulated to contain 14.10 and 16.06% DM intake to test the effect of SG containing saponin and tannins on nitrogen utilization efficiency. SG is composed of 162 g/kg DM of saponin and 108.7 g/kg DM of tannin.

### 3.2. Feed Intakes and Digestibility Coefficients

The influence of CP content in concentrate and SG supplementation on feed intake and apparent digestibility is presented in Table 3. The CP content in concentrate and supplementation of SG had no interaction effects on DM intake and nutrient digestibility. Increasing CP content in concentrate did not influence DM intake and nutrient digestibility. SG supplementation significantly (*p* < 0.05) affected the CP digestibility, although the response of others did not differ. Increasing SG supplementation to 0.6% significantly decreased CP digestibility, 5.7% lower than with 0.4% supplementation.

### 3.3. pH, Ammonia Nitrogen, Protozoa, and Blood Urea Nitrogen

The influence of CP content in concentrate and SG supplementation on ruminal pH, NH_3_-N, protozoal count, and BUN is shown in Table 4. The CP content in concentrate and SG supplementation had no interaction effects on pH, NH_3_-N, protozoal number, and BUN. Increasing the CP content in concentrate to 16% significantly (*p* < 0.05) increased the ruminal NH_3_-N concentration at 4 h post-feeding, 5.05% higher than with 14% CP in concentrate. The average concentration of ruminal NH_3_-N was 19.06 mg/dL for 14% CP and 19.88 mg/dL for 16% CP in concentrate (Table 4). Increasing the SG supplementation to 0.6% significantly (*p* < 0.05) decreased ruminal NH_3_-N concentration, protozoal number, and BUN by 12%, 45%, and 9%, respectively, at 4 h post-feeding.

### 3.4. Ruminal Volatile Fatty Acids and Methane Estimation

The influence of CP content in concentrate and SG supplementation on total VFA, C2, C3, C4, C2:C3 ratio, and CH_4_ estimation is shown in Table 5. The CP content in concentrate and SG supplementation had no interaction effects on total ruminal VFA and their proportions and CH_4_ production. The CP content in concentrate did not affect the ruminal fermentation products and CH_4_ estimation. Increasing SG supplementation significantly affected total VFA, C2, C3, C2:C3 ratio, and CH_4_ production, although C4 was not affected. A 0.6% supplementation of SG significantly increased the average total VFAs and C3 concentration by 0.84% and 7.13%, respectively, compared with 0.4% supplementation. In contrast, 0.6% supplementation of SG significantly decreased the average C2, C2:C3 ratio, and CH_4_ production by 2.71%, 13.17%, and 4.37%, respectively, compared with 0.4% supplementation.

### 3.5. Nitrogen Utilization

The influence of CP content in concentrate and SG supplementation on nitrogen (N) intake, N excretion, N absorption, and N retention is shown in Table 6. The CP content in concentrate and SG supplementation had no interaction effects on N utilization. The CP content in concentrate significantly (*p* < 0.05) influenced N intake, absorption, and retention (Table 6). Increasing the CP content increased N intake, absorption, and retention. SG supplementation significantly influenced total N excretion and fecal N excretion; the responses of others did not differ. Increasing the SG supplementation to 0.6% significantly decreased total N excretion and fecal N excretion by 13% and 14%, respectively.

## 4. Discussion

### 4.1. Feed Intakes and Digestibility Coefficients

The CP content in concentrate and SG supplementation had no interaction effects on feed intake and digestibility of nutrients. The CP content in concentrate did not affect total DM intake and nutrient digestibility. National Research Council (NRC) [21] demonstrated that nutrient digestibility has a positive relationship with DM intake. Thus, nonsignificant nutrient digestibility might be due to the nonsignificant effect of CP levels on DM intake. Norrapoke et al. [22] similarly found that feeding concentrate containing 16% and 19% of CP did not affect the nutrient digestibility. Aguerre et al. [23] evaluated two CP levels at 15.3% and 16.6% in dairy cows, resulting in no effect on nutrient digestibility. SG supplementation did not influence intake and nutrient digestibility, except CP digestibility. SG supplementation at 0.6% of DM intake (23.48 g/d of tannin and 34.99 g/d of saponins) significantly decreased the CP digestibility. This could be due to the tannin content in SG that could prevent ingested protein digestion in the rumen via tannin–protein complex formation [7]. In addition, 0.6% SG supplementation did not affect DM, OM, NDF, and ADF digestibility, suggesting cattle that consumed 23.48 g/d did not affect the ruminal microbial activity. Gutierrez et al. [24] stated that saponin–tannins binding could relieve saponin suppression of bacterial activity and ruminal degradation of saponin. Rira et al. [6] suggested that this may act as a substrate for tannins chelator [25]. Consuming chemical chelators like tannins and other toxic compounds like saponin could reduce the toxicity produced by tannins [26]. Aguerre et al. [23] found that cows fed diets containing tannin extract at 0%, 0.45%, 0.9%, and 1.8% of dietary DM (106.65, 214.2, and 397.8 g/d of tannins) linearly decreased DM, OM, CP, and NDF digestibility compared with the control. Decreasing nutrient digestibility including DM, OM, and NDF showed a negative effect of high consumption of tannins. Hassanat and Benchaar [27] found that 5% hydrolyzed tannins from chestnut or 5% condensed tannins from acacia decreased in vitro ruminal protein digestibility. Cherdthong et al. [4] similarly found that cattle fed with *Delonix regia* seed meal containing 0.085% tannins and 1.1% of saponins at 150 g/day (0.13 g/d of tannin and 1.1 g/d of saponin) showed no effect on nutrient digestibility; this might be due to the lower tannin consumption compared with the current study. Guyader et al. [28] found a diet containing tea saponin extract containing 68.9% of saponin at 0.76% of dietary DM (92.16 g/d) fed to dairy cows did not influence the nutrient digestibility. Makkar et al. [29] stated that saponins might either affect or have no effect on nutrient digestibility. Holtshausen et al. [30] revealed that inclusion of *Yucca schidigera* containing 6% of saponins and *Quillaja saponaria* comprising 3% of saponins powder into a total mixed ration at 10 g/kg DM did not influence nutrient digestibility. This discrepancy for CP digestibility could relate to many factors including supplement sources, sources’ form, dose study, and composition of diet [24,31].

### 4.2. pH, Ammonia Nitrogen, Protozoa, and Blood Urea Nitrogen

The CP content in concentrate and SG supplementation did not show any interaction effects on pH, NH_3_-N, protozoal number, and BUN (Table 4). Increasing CP content in concentrate significantly increased the ruminal NH_3_-N concentration. The NH_3_-N concentration was 19.06 mg/dL for 14% CP and 19.88 mg/dL for 16% CP in concentrate. The greater NH_3_-N concentration with 16% CP in concentrate suggested that more protein entered the rumen, which allowed microbes to convert it into NH_3_-N, when compared with 14% CP in concentrate. Similarly, Ampapon and Wanapat [32] found that increasing CP levels (14%, 16%, and 18%) in concentrate affected the in vitro ruminal NH_3_-N concentration. Norrapoke et al. [22] showed that protein levels (16 vs. 19%) did not affect the ruminal NH_3_-N concentration of lactating dairy cows, but significantly affected the BUN concentration according to Campanile et al. [33], who also showed that CP intake and CP quality, i.e., durability, affect BUN. SG supplementation significantly decreased NH_3_-N, BUN, and protozoal numbers. The decrease of NH_3_-N concentration with 0.6% of SG supplementation could be due to the protection of protein metabolism in the rumen by tannins via tannin–protein complex formation, while the lower BUN concentration could be caused by the decrease of NH_3_-N concentration. A similar finding was reported by Bhatta et al. [30], who found a significant decrease of in vitro NH_3_-N concentration with increased levels of trees (*Autocarpus integrifolis*, *Azardirachta indica*, and *Ficus bengalensis*) containing PSCs. Furthermore, Holtshausen et al. [30] revealed that feeding saponin-containing *Yucca schidigera* and *Quillaja Saponaria* quadratically decreased in vitro NH_3_-N concentration compared with the control. SG supplementation significantly decreased the protozoal number at 4 h post-feeding. This result was in agreement with previous studies: Norrapoke et al. [22] used mangosteen peel pellets containing 14.6% of condensed tannins and 9.5% of saponins at 0 and 300 g/d in lactating dairy cows; Ampapon and Wanapat [32] used rambutan peel powder comprising 1.20% of condensed tannins and 1.03% of saponins at 0%, 2%, 4%, and 6% of dietary DM in an in vitro study; and Cherdthong et al. [4] used *Delonix regia* seed meal (*Delonix regia*) comprising 0.085% of tannins and 1.10% of saponins at 0, 50, 100, and 150 g/d in Thai native beef cattle. The reduction of protozoal number could be due to the presence of saponins and tannins in the SG. Saponins have been reported to be toxic to protozoa [34]. Regarding the mechanism of saponins on protozoa, it has been proposed by Makkar et al. [34] that saponins may form complexes with a lipid membrane, which increases permeability, causes an imbalance, and consequently promotes cell lysis. Wallace et al. [35] proposed that saponins form complexes with sterols on the surface of the protozoal membrane, which causes impairment and disintegration. Tannins can directly affect methanogen bacteria, but not for protozoa [36]. They may indirectly affect protozoal number [36], and a possible mechanism has been proposed by Tavendale et al. [37]. That is, tannins may bind to proteinaceous adhesin or part of the cell envelope of methanogenic archaea, which impairs the methanogen–protozoa complex formation, decreases interspecies hydrogen transfer, and inhibits methanogen growth. Methanogenic archaea and protozoa have a symbiotic association [36]; thus, tannins indirectly affect the protozoal number.

### 4.3. Ruminal Volatile Fatty Acid Profiles and Methane Estimation

The interaction effect between CP content in concentrate and SG supplementation was not found for total VFA, C2, C3, C4, C2:C3 ratio, and CH_4_ estimation. An increase in the SG supplementation significantly increased the total VFA and C3 concentration, while significantly decreasing the C2:C3 ratio and CH_4_ estimation. Total VFA production is closely related to the VFA proportions, including C2, C3, and C4. A reduction in the total VFA occurred with a change in the VFA proportions, such as an increase in C2 and decrease in C3 [38,39]. In this study, the increase in SG supplementation significantly decreased C2 concentration. Similarly, Beauchemin et al. [40] found a decrease of C2 with feed including quebracho containing tannins, and Castro-Montoya et al. [41] found a diet containing mimosa, sumach, and chestnut resulted in a decrease in C2 concentration. Saponins’ effects on VFA products vary depending on the studied dose. An increase in SG supplementation significantly increased C3 concentration. This could be related to the decrease in the protozoal number and CH_4_ production, providing more available hydrogen for C3 synthesis. Similar results have been reported [41]. Saponins may inhibit acetate producers and protozoa and may favor propionate-producing bacteria, resulting in greater C3 concentration in the rumen [35]. The CH_4_ emission was mitigated by SG supplementation. A reduction of CH_4_ production by saponins and tannins had been widely reported [3,4,8,22]. Saponins have been revealed to mitigate CH_4_ production and change ruminal fermentation [8]. The reduction of CH_4_ production could be linked to the reduction of the protozoal number and C2:C3 ratio. Reduction of the C2:C3 ratio provided more hydrogen for C3 production, resulting in less hydrogen for methanogenesis and subsequently reduced CH_4_ production. Protozoa can provide hydrogen for the methanogen bacteria and act as the host for methanogen [31], thus lowering the protozoal number with a reduction of CH_4_ production. Tannins can directly affect methanogenesis by suppressing ruminal archaea, but not removing protozoa [36]. As methanogens attach to protozoa, tannins’ effect on protozoa could decrease CH_4_ production because protozoa can synergistically offer hydrogen for the methanogenesis process [31].

### 4.4. Nitrogen Utilization

Interaction between CP content in concentrate and SG supplementation did not affect intake, excretion, absorption, and retention of nitrogen. The N intake, absorption, and retention were increased when CP content increased. The increase in N intake might be due to the increase in CP content in the concentrate, and subsequently resulted in a greater N absorption and retention. Aguerre et al. [23] found that two dietary CP levels at 15.3% and 16.6% fed to dairy cows did not affect N intake, this might be due to the different interval between 15.3% and 16.6% CP being small to meet the statistical significance. Increasing SG supplementation to 0.6% significantly decreased total N excretion and N excretion in feces. However, SG supplementation did not affect either N absorption or retention. The decrease in total N and fecal N excretion with SG supplementation suggested that the tannin–protein complex might disassociate at the abomasum, meaning that post-ruminal protein metabolism was enhanced. Thus, protein digestibility at the small intestine would be determined. Cherdthong et al. [4] similarly revealed that royal poinciana seed meal pellet (comprising 0.085% of tannins and 1.10% of saponins) up to 150 g/d fed to beef cattle quadratically significantly decreased fecal N excretion. The lower fecal N excretion could also be due to the lower NH_3_-N concentration in the rumen (Table 4) when SG supplementation increased. Tannins have been widely reported for their effect on slowing down the degradation rate of protein in the rumen [33] via tannin–protein complex formation at the ruminal pH and subsequently reduced NH_3_-N concentration in the rumen. The tannin–protein complexes’ disassociation at the abomasum post escaping ruminal fermentation affected the N excretion [42]. However, some studies have reported higher fecal N excretion with diets containing tannins [36,43]. Aguerre et al. [23] revealed that increasing tannins extract from 0% to 1.18% of dietary DM linearly increased fecal N excretion. The higher fecal N excretion could be explained by the fact that tannin–protein complexes were not completely disassociated at the abomasum and subsequent digestive tracts [43]. The ability of tannins to bind to protein varied depending on the sources and chemical properties, such as molecular weight, and the post-ruminal disassociation process between tannins and protein [44,45] would thus be different, which may have caused the contrasting finding in terms of fecal N excretion compared with previous studies.

## 5. Conclusions

From this study, the CP content in concentrate and SG supplementation did not have an interaction effect on feed utilization, ruminal ecology, ruminal fermentation products, and N utilization. Increasing the CP content in concentrate significantly increased NH_3_-N concentration and N intake, absorption, and retention. SG supplementation at 0.6% showed a greater total VFAs and C3 concentration, and decreased CP digestibility, NH_3_-N concentration, BUN concentration, protozoal number, C2:C3 ratio, CH_4_ production, and fecal N excretion when compared with the 0.4% supplementation. The authors would recommend supplementing SG at 0.6% to the concentrate-based diet containing either 14% or 16% CP.

## Figures and Tables

**Table 1:** Feed ingredients and chemical composition of concentrate diet containing various crude protein (CP), rice straw, and *S. grandiflora* pods meals (SG). DM, dry matter.

Items	Concentrate		Rice Straw	SG
	14% CP	16% CP		
Ingredient, % dry matter				
Cassava chip	53.0	52.5	-	-
Soybean meal	12.5	16.5	-	-
Rice bran	15.0	12.0	-	-
Palm kernel meal	14.0	13.5	-	-
Urea	1.6	1.6	-	-
Premix *	1.0	1.0	-	-
Molasses	1.4	1.4	-	-
Sulfur	0.5	0.5	-	-
Salt	1.0	1.0	-	-
Chemical composition, %				
Dry matter, %	91.06	91.43	92.20	94.19
Organic matter, %DM	84.97	86.10	90.61	93.86
Crude protein, %DM	14.10	16.06	3.27	22.48
Ether extract, %DM	2.05	2.17	1.46	4.42
Neutral detergent fiber, %DM	30.87	28.76	71.74	56.67
Acid detergent fiber, %DM	13.57	12.68	47.73	43.12
Gross energy (GE), MJ/kg DM	4.06	4.14	3.83	-
Condensed tannin, (g/kg DM)	-	-	-	108.7
Saponins, g/kg DM	-	-	-	162.0

* Minerals and vitamins (each kg contains): vitamin A: 10,000,000 IU; vitamin E: 70,000 IU; vitamin D: 1,600,000 IU; Fe: 50 g; Zn: 40 g; Mn: 40 g; Co: 0.1 g; Cu: 10 g; Se: 0.1 g; I: 0.5 g.

**Table 2:** Chemical compositions of the rations based on dry matter.

Items	14% CP		16% CP	
	0.4% SG	0.6% SG	0.4% SG	0.6% SG
Dry matter, %	91.81	91.74	91.81	91.72
Organic matter, %	88.49	88.51	88.50	88.43
Crude protein, %	7.97	7.86	7.94	8.02
Ether extract, %	1.76	1.76	1.76	1.77
Neutral detergent fiber, %	55.44	55.98	55.52	55.39
Acid detergent fiber, %	34.31	34.79	34.37	34.31
Gross energy, MJ/kg DM	3.82	3.80	3.82	3.81
Condensed tannin, %	0.27	0.31	0.27	0.31
Saponin, %	0.40	0.46	0.40	0.46

CP = crude protein, SG = *S. grandiflora* pods meals.

**Table 3:** Effects of crude protein (CP) levels (14 vs. 16%) in concentrate with *S. graniflora* pods meal (SG, 0.4% vs. 0.6% dry matter intake) on feed intake and digestibility in Thai native beef cattle.

Item	14% CP		16% CP		CP		SG		SEM	*p*-Value		
	0.4% SG	0.6% SG	0.4% SG	0.6% SG	14%	16%	0.4%	0.6%		CP	SG	CP × SG
Dry matter intake												
Roughage intake, kg/d	2.36	2.43	2.38	2.36	2.395	2.37	2.37	2.395	0.06	0.862	0.844	0.734
%BW	1.57	1.63	1.55	1.55	1.60	1.55	1.56	1.59	0.03	0.489	0.631	0.613
g/kg BW^0.75^	54.88	57.06	54.68	54.53	55.97	54.605	54.78	55.795	1.17	0.572	0.673	0.630
Concentrate intake, kg/d	1.57	1.52	1.57	1.57	1.545	1.57	1.57	1.545	0.03	0.712	0.712	0.712
%BW	1.05	1.03	1.03	1.04	1.04	1.03	1.04	1.035	0.02	0.891	0.872	0.729
g/kg BW^0.75^	36.63	35.89	36.21	36.38	36.26	36.29	36.42	36.13	0.55	0.976	0.801	0.688
SG, kg/d	0.100	0.116	0.100	0.116	0.11	0.11	0.10	0.12	0.01	1.000	0.068	1.000
Total intake kg/d	4.03	4.07	4.05	4.05	4.05	4.05	4.04	4.06	0.09	0.991	0.931	0.918
%BW	2.68	2.74	2.65	2.67	2.71	2.66	2.665	2.705	0.05	0.573	0.678	0.804
g/kg BW^0.75^	93.83	95.69	93.19	93.60	94.76	93.39	93.51	94.64	1.60	0.676	0.729	0.824
Nutrient digestibility, %												
Dry matter	65.82	65.52	67.56	69.27	65.67	68.41	66.69	67.39	4.40	0.395	0.825	0.752
Organic matter	69.32	69.15	70.84	71.60	69.23	71.22	70.08	70.37	2.48	0.281	0.866	0.796
Crude protein	70.01	67.82	73.16	67.62	68.915	70.39	71.58 ^a^	67.72 ^b^	1.90	0.296	0.014	0.239
Neutral detergent fiber	68.46	69.48	68.52	67.39	68.97	67.95	68.49	68.43	2.01	0.490	0.969	0.468
Acid detergent fiber	55.44	59.20	55.40	56.50	57.32	55.95	55.42	57.85	3.44	0.585	0.338	0.595

CP × SG = interaction between CP and *S. graniflora* pods meal, BW = body weight, SEM = standard error of the mean. ^ab^ means with different superscript letter within rows were significantly different at *p* < 0.05.

**Table 4:** Effects of crude protein (CP) levels (14% vs. 16%) in concentrate with *S. graniflora* pod meal (SG, 0.4% vs. 0.6% dry matter intake) on ruminal pH, ammonia nitrogen, ruminal protozoal population, and blood urea nitrogen concentration in Thai native beef cattle.

Item	14% CP		16% CP		CP		SG		SEM	*p*-Value		
	0.4% SG	0.6% SG	0.4% SG	0.6% SG	14%	16%	0.4%	0.6%		CP	SG	CP × SG
pH												
0 h pre-feeding	6.76	6.80	6.77	6.81	6.78	6.79	6.76	6.80	0.16	0.933	0.738	0.983
4 h post-feeding	6.59	6.71	6.62	6.70	6.65	6.66	6.60	6.70	0.09	0.850	0.148	0.762
Mean	6.67	6.76	6.70	6.76	6.715	6.73	6.68	6.76	0.13	0.860	0.582	0.910
Ammonia nitrogen, mg/dL												
0 h pre-feeding	15.96	16.32	16.26	16.95	16.14	16.61	16.11	16.64	0.56	0.266	0.208	0.687
4 h post-feeding	23.43	20.54	24.42	21.89	21.99 ^b^	23.16 ^a^	23.93 ^a^	21.22 ^b^	0.69	0.013	<0.001	0.394
Mean	19.70	18.43	20.34	19.42	19.07 ^b^	19.88 ^a^	20.02 ^a^	18.93 ^b^	0.42	0.008	0.003	0.334
Protozoa, ×10^5^ cell/mL												
0 h pre-feeding	7.01	6.70	7.25	7.02	6.86	7.14	7.13	6.86	0.47	0.288	0.309	0.866
4 h post-feeding	10.69	7.15	10.60	7.49	8.92	9.05	10.65 ^a^	7.32 ^b^	0.62	0.783	<0.001	0.637
Mean	9.13	6.49	9.31	6.96	7.81	8.14	9.22 ^a^	6.73 ^b^	0.27	0.251	<0.001	0.905
Blood urea nitrogen concentration, mg/dL												
0 h pre-feeding	10.28	10.39	10.76	11.05	10.34	10.91	10.52	10.72	0.40	0.087	0.492	0.760
4 h post-feeding	12.10	11.33	12.44	11.15	11.72	11.80	12.27 ^a^	11.24 ^b^	0.55	0.838	0.021	0.522
Mean	11.19	10.86	11.60	11.10	11.03	11.35	11.40	10.98	0.32	0.069	0.224	0.713

CP × SG = interaction between crude protein and *S. graniflora* pod meal, SEM = standard error of the mean. ^ab^ means with different superscript letter within rows were significantly different at *p* < 0.05.

**Table 5:** Effects of crude protein (CP) levels (14% vs. 16%) in concentrate with *S. graniflora* pod meal (SG, 0.4% vs. 0.6% dry matter intake) on volatile fatty acid profile and methane estimation in Thai native beef cattle.

Item	14% CP		16% CP		CP		SG		SEM	*p*-Value		
	0.4% SG	0.6% SG	0.4% SG	0.6% SG	14%	16%	0.4%	0.6%		CP	SG	CP × SG
Total volatile fatty acid, mmol/L												
0 h pre-feeding	100.60	101.34	100.70	101.32	100.97	101.01	100.65	101.33	0.54	0.913	0.105	0.873
4 h post-feeding	105.66	106.63	106.66	107.87	106.15	107.27	106.16	107.25	0.68	0.278	0.288	0.902
Mean	103.13	103.98	103.68	104.59	103.56	104.14	103.41 ^b^	104.29 ^a^	0.69	0.456	0.013	0.830
Volatile fatty acid, profiles, %												
Acetic acid												
0 h pre-feeding	65.14	63.92	65.10	63.68	64.53	64.39	65.12	63.80	0.55	0.779	0.061	0.384
4 h post-feeding	67.43	65.40	67.08	64.76	66.42	65.92	67.26 ^a^	65.08 ^b^	0.51	0.065	<0.001	0.961
Mean	66.28	64.66	66.09	64.22	65.47	65.16	66.19 ^a^	64.44 ^b^	0.41	0.307	<0.001	0.224
Propionic acid												
0 h pre-feeding	21.40	22.31	21.08	22.63	21.86	21.86	21.24	22.47	0.60	0.393	0.182	0.659
4 h post-feeding	22.61	24.42	23.01	25.50	23.52	24.26	22.81 ^b^	24.96 ^a^	0.84	0.420	<0.001	0.853
Mean	22.00	23.37	22.05	24.06	22.69	23.06	22.03 ^b^	23.72 ^a^	0.55	0.890	<0.001	0.975
Butyric acid												
0 h pre-feeding	13.45	13.76	13.80	13.68	13.61	13.74	13.63	13.72	0.53	0.504	0.567	0.671
4 h post-feeding	10.22	10.17	9.90	9.73	10.20	9.82	10.06	9.95	0.62	0.574	0.595	0.893
Mean	11.84	11.96	11.85	11.70	11.90	11.78	11.85	11.83	0.43	0.329	0.311	0.522
Acetic acid to propionic acid	3.01	2.71	3.09	2.68	2.86	2.89	3.05 ^a^	2.70 ^b^	0.06	0.760	<0.001	0.419
Methane estimation, mmol/L												
0 h pre-feeding	28.81	28.13	29.02	27.91	28.47	28.47	28.92	28.02	0.55	0.984	0.413	0.588
4 h post-feeding	27.77	26.62	27.60	25.80	27.20	26.70	27.69 ^a^	26.21 ^b^	0.43	0.209	<0.001	0.208
Mean	28.29	27.38	28.31	26.85	27.84	27.58	28.30 ^a^	27.12 ^b^	0.35	0.409	<0.001	0.223

CP × SG = interaction between crude protein and *S. graniflora* pod meal, BW = body weight, SEM = standard error of the mean. CH_4_ estimation= (0.45 ×acetic acid) − (0.275 × propionic acid) + (0.40 × butyric acid) [19]. ^ab^ means with different superscript letter within rows were significantly different at *p* < 0.05.

**Table 6:** Effects of crude protein (CP) levels (14% vs. 16%) in concentrate with *S. graniflora* pod meal (SG, 0.4% vs. 0.6% dry matter intake) on nitrogen (N) balance in Thai native beef cattle.

Item	14% CP		16% CP		CP		SG		SEM	*p*-value		
	0.4% SG	0.6% SG	0.4% SG	0.6% SG	14%	16%	0.4%	0.6%		CP	SG	CP × SG
N intake, g/d	51.47	51.27	56.52	56.98	51.37 ^b^	56.75 ^a^	54.00	54.13	1.09	0.030	0.953	0.882
N excretion, g/d	16.06	13.70	15.95	14.21	14.88	15.08	16.01 ^a^	13.96 ^b^	0.38	0.795	0.019	0.691
Fecal N excretion, g/d	13.36	11.42	13.09	11.77	12.39	12.43	13.23 ^a^	11.60 ^b^	0.34	0.951	0.032	0.656
Urinary N excretion, g/d	2.70	2.28	2.86	2.44	2.49	2.65	2.78	2.36	0.11	0.474	0.076	0.998
N absorption, g/d	38.11	39.85	43.43	45.22	38.98 ^b^	44.33 ^a^	40.77	42.54	1.02	0.022	0.404	0.992
N retention, g/d	35.41	37.57	40.57	42.77	36.49 ^b^	41.67 ^a^	37.99	40.17	1.06	0.031	0.325	0.992

CP × SG = interaction between crude protein and *S. graniflora* pod meal, SEM, standard error of the mean. ^ab^ means with different superscript letter within rows were significantly different at *p* < 0.05.

## Data Availability

Not applicable.
